# Global Diseases Deserve Global Solutions: Alzheimer’s Disease

**DOI:** 10.3390/neurolint17060092

**Published:** 2025-06-14

**Authors:** Emma Twiss, Carley McPherson, Donald F. Weaver

**Affiliations:** 1 Krembil Research Institute, University Health Network, Toronto, ON M5T 0S8, Canada; emma.twiss@queensu.ca; 2 Lakeridge Health, Oshawa, ON L1G 8A2, Canada; camcpherson@lh.ca; 3 Departments of Medicine (Neurology) and Chemistry, University of Toronto, Toronto, ON M5S 1A1, Canada

**Keywords:** Alzheimer’s disease, dementia, risk factors, global emergency, disease prevention

## Abstract

Alzheimer’s Disease (AD) is a global issue, with increasing incidence and prevalence as the world’s population ages and life expectancy increases. Projections indicate that by 2050, over 150 million individuals worldwide will be personally living with AD, an impending crisis made worse by the absence of cure therapies. Moreover, the risk factor relationship of dementia with rising global temperatures and air pollution further necessitates the urgency of a coordinated international response. With an extensive economic and emotional burden, AD is no longer just a disease; it is a worldwide societal crisis. This review presents five calls to action to address the AD global health emergency. First, AD research must be approached as an internationally performed activity, involving standardized data sharing, collaborative innovation, and improved access to pharmaceutical studies in low- and middle-income countries (LMICs), alongside increased diversity, inclusion, and equity in research. Second, there must be a commitment to develop universally accessible, affordable, and non-invasive diagnostic tools for AD. Third, advancements in AD therapeutics should prioritize the development of affordable agents, allowing for widespread geographic distribution. Fourth, we identify focus areas for global dementia risk reduction: sleep, head injury prevention, exercise, learning, and diet (SHIELD risk reduction strategy). Fifth, improving care for individuals with AD requires eliminating stigma through educational programs for both the public and caregivers. The escalating AD crisis demands an unprecedented global coalition in research, diagnostics, therapeutics, prevention, and education to avoid a future where the disease becomes the defining crisis of our era.

## 1. Introduction

Global life expectancy at birth has risen dramatically over the past half-century, from approximately 38 years in 1950 to 73 years in 2022 [1,2]. With this sharp increase in life expectancy has come the expectation that not only will we live longer, but also healthier—an expectation that applies globally, beyond wealthy nations [3]. The quest for a healthier, longer life on a global scale must address the myriads of age-related illnesses that accompany enhanced longevity, with Alzheimer’s Disease (AD; and related dementias [ADRD]) being one of the most common age-associated diseases of worldwide prevalence.

AD is the most common type of dementia, involving the pathological aggregation of misfolded β-amyloid fibrils and tau proteins within brain [4]. AD should not be regarded as an inevitable consequence of aging; yet, according to the Centers for Disease Control and Prevention (CDC), with each five-year increment past age sixty-five, the risk of developing AD doubles. Indeed, every three seconds, a new case of AD emerges globally [5]. In accord with this statistic, the World Health Organization (WHO) estimates that the prevalence of AD has surged by 148% from 1990 to 2019, currently impacting an estimated 55 million individuals worldwide [6]. This situation can be attributed to the irreversible nature of AD and the lack of a cure to treat its devastating impact. During the 2013 G8 Dementia Global Summit, it was declared that a cure or disease-modifying therapy for dementia must be on the market by 2025 to avoid devastating health and socioeconomic consequences on a worldwide scale [7]. Though this deadline is now at hand, the goal of a cure remains frustratingly distant.

This review does not aim to suggest definitive solutions to the AD crisis, but instead presents a narrative that emphasizes the need for urgent global research and action. By illustrating the current dire situation, the socioeconomic burden, and evaluating lessons from global health responses such as the COVID-19 pandemic, we advocate for a coordinated global approach to combating AD. We propose five key commitments to reduce the AD burden: enhancing international coordination in AD research, advancing diagnostic tool development, focusing on innovative therapeutics research, promoting preventative measures, and improving the care for those with dementia worldwide. Essential to these efforts remains the needs to reduce the number of people developing dementia and to discover a cure—a commitment owed to those currently living with AD and to the memory of those we have lost. The time for collaborative action is now.

## 2. AD as a Global Crisis

### 2.1. The AD Emergency

With AD cases on the rise globally, two parallel threats are concomitantly evolving as dementia risk factors: climate change and air pollution. Collectively, these crises are synergistically creating a dangerous brain–health maelstrom that necessitates a unified global response to prevent a healthcare crisis. In light of the escalating global burden of the disease, continued inaction is increasingly difficult to justify. While further evaluative studies have their place, overreliance on calls for additional assessment may impede progress. A balanced approach that integrates ongoing evaluation with timely, evidence-informed action is now essential.

**Figure 1:**
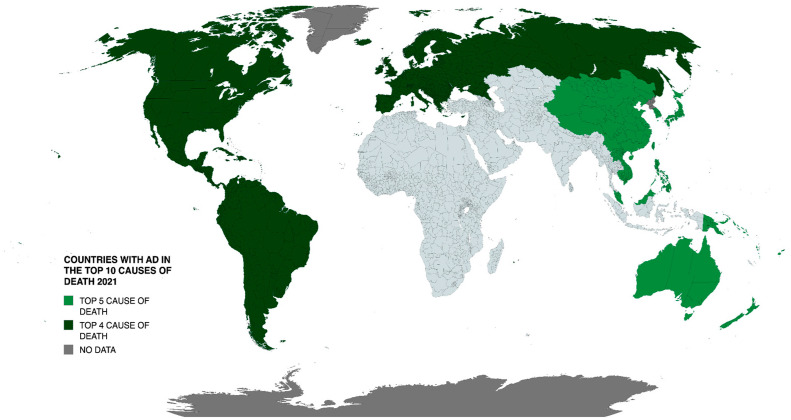
Geographic distribution of countries where AD and related dementias ranked among the top 10 causes of death in 2021. Data sourced from WHO World Health Statistics 2024.

AD is currently the seventh leading cause of death worldwide, yet receives just 1.5% of global health research funding (reported by the WHO). Figure 1 illustrates the global distribution whereby AD and related dementias rank amongst the top ten causes of death. Projections suggest that by 2050, AD cases could exceed 131 million or more, eventually representing one in every three people over age 85 years [7,8]. With these projections comes the expectation that the number of deaths attributed to AD will also rise, creating a situation that will have a catastrophic impact on older and younger generations.

The overlap between AD and environmental factors is becoming increasingly evident. Climate change and severe air pollution exacerbate the prevalence and severity of AD, particularly in low- and middle-income countries (LMICs); rising temperatures and environmental disasters heighten health risks, putting individuals with dementia at higher risk as they are more susceptible to cognitive decline due to increased stress on brain health [9]. Every one °C increase above 17 °C in ambient environmental temperature is associated with a 4.5% rise in hospital admissions among people with dementia, leading to increased mortality rates and accelerated cognitive decline post hospitalization [9,10,11]. Moreover, atypical weather patterns contribute to heightened social isolation, disrupted healthcare access, and increased stress, further accelerating cognitive decline in affected individuals [12].

Additional cases of dementia are directly related to high levels of harmful airborne pollutants such as fine particulate matter (PM2.5), nitrogen dioxide (NO₂), and carbon monoxide (CO) [13,14]. These miniscule particles can bypass the blood–brain barrier and deposit in the brain, resulting in enhanced neuroinflammatory processes linked to dementia progression [15,16]. Daily exposure to PM2.5, such as through traffic-related emissions, can lead to significant neurological damage attributed to a decrease in cerebrospinal fluid (CSF) Ab_42_ [17]. These levels paradoxically reflect increased β-amyloid plaque accumulation in the brain—these plaques can accumulate years before cognitive symptoms arise. Higher emissions of PM2.5, such as from wildfires, pose a 21% increased risk of dementia for every increase in 1 µg/m^3^ of PM2.5 in the air [18]. The wildfire situation increasingly worsens with each passing year as wildfires burn double the landscape compared to twenty years ago, according to the World Resources Institute. Given that air pollution affects 99.82% of the Earth’s land surface, this issue presents a pervasive threat [18]. The escalating AD cases, compounded by climate change and air pollution, create a costly and escalating emergency demanding immediate action.

### 2.2. The Economic and Societal Burden of AD

**Figure 2:**
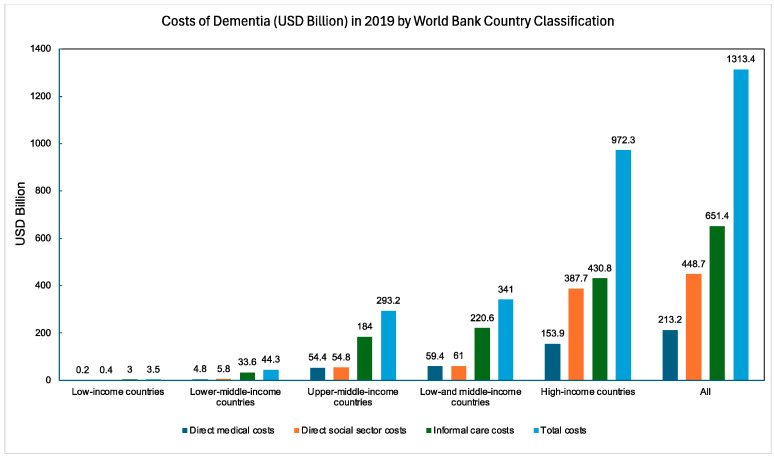
Global dementia costs (USD billion) in 2019 categorized by the World Bank Country Classification [19].

AD is not solely a biomedical issue—it is also a socioeconomic issue. In 2019, the global costs related to AD, encompassing care expenses, medications, and other health-related expenditures, amounted to an estimated USD 131.4 billion for 55.2 million cases [19]. By 2030, these costs are projected to increase to USD 4.7 trillion, although this figure is underestimated due to limited studies in LMICs [20,21]. AD is a slow and progressive disease that necessitates intensive caregiving as it progresses. The total cost of caring for an individual with AD from diagnosis to death is estimated at USD 412,936, with 70% of these costs being borne by families [22]. In 2022, 11.3 million informal caregivers of individuals with AD provided 16 billion hours of unpaid assistance—271.6 billion USD worth of care [22]. Figure 2 depicts the societal and informal costs of AD across different countries, including lost wages.

The full economic burden remains misunderstood and inadequately studied. The bulk of the costs (direct, indirect, hidden) stem from caring for individuals with AD, and even these numbers fail to capture the emotional toll that AD exerts on families. Family and caregivers cope with the gradual loss of a loved one, alongside their own health challenges, including elevated risks of stress-related illnesses such as obesity, cancer, and depression [22]. In the future, a 35% increase in the number of caregivers will be necessary by 2028 to meet the projected demand, a concerning statistic given the prevalence of stress-related burnout in caregivers [22]. The global economic and societal toll in caring for individuals with AD further reflects the need for effective solutions.

### 2.3. Lessons from the COVID-19 Pandemic

The COVID-19 pandemic offers insightful lessons for addressing AD, as both crises disproportionately impact older individuals and LMICs [23]. The global response to the pandemic served as a reminder of the need for equitable access to healthcare and innovative solutions, such as temporary waivers of intellectual property rights to expedite vaccine access [24].

The rollout of COVID-19 vaccines created significant challenges, including disparities in vaccine distribution favoring high-income countries. A WHO-endorsed “COVAX” program was created during the pandemic to accelerate the production of COVID-19 vaccines by sharing global data, with the goal of distributing vaccines to two-thirds of the world’s countries. This was not the outcome, as high-income countries received the vaccine first, leaving LMICs with fewer resources [25]. This situation further emphasizes the need for global health equity and the unbiased sharing of resources to combat the common goal of AD, and not leaving LMICs, where there are the most AD cases, with fewer resources.

The COVID-19 pandemic also demonstrated the importance of effective communication strategies, with social media campaigns and telehealth being heavily relied upon for information dissemination. Specifically, vaccine social media campaigns proved to be beneficial during the pandemic as they increased the number of people willing to be immunized [26]. However, with the increased use of social platforms, there must be extreme caution regarding the concomitant spread of misinformation. Using social media to increase awareness of AD, such as through the sharing of information about risk factors and the use of verified sources to obtain health information, can raise awareness and care about the disease. Along the same lines, the COVID-19 pandemic heavily relied upon “telehealth”, i.e., the act of providing health services virtually [27]. The use of telehealth can similarly help people with AD globally, connecting them to healthcare workers in different regions and potentially improving their treatment options [28].

The pandemic served as a necessary reminder of the critical role of healthcare workers. During the pandemic, there were unprecedented levels of burnout and stress among healthcare workers [29]. As there already exists high levels of caregiver stress amidst those caring for individuals with AD, the importance of support services for the physical and mental well-being of these workers must not be understated [30]. As the AD situation increasingly calls for more caregivers, this lesson must not be forgotten for the health and safety of both patients and caregivers. Overall, the COVID-19 pandemic has provided a lesson for global solidarity and innovation in tackling complex health challenges such as AD [31].

## 3. Five Calls to Action

Herein, the implementation of five calls to action on a worldwide scale is proposed, with the explicit goals of 1. advancing research globally, 2. identifying diagnostic tools for worldwide implementation, 3. discovering disease modifying therapeutics that are affordable and accessible, 4. preventing dementia by risk reduction at a global level, and 5. bettering the care for those living with its effects.

### 3.1. AD Research as a Global Enterprise

Commitment: Global cooperation on all aspects of AD research through unified data sharing, improving access to pharmaceuticals and expanding research diversity.

#### 3.1.1. Unified Data Sharing and Collaborative Innovation

For there to be advances in all aspects of AD research, global coordination is necessary. Collaborations amongst stakeholders and robust data-sharing mechanisms are essential; public funding cannot be relied upon alone [7,32]. Data sharing drives scientific progress, minimizes research fraud, and allows for the widespread distribution of knowledge [33]. Research efforts are more effective if data from AD studies are linked by a worldwide network, allowing improved data access. Initiatives such as the “Global Alzheimer’s Association Interactive Network” (GAAIN) and Integrated Neurodegenerative Disease Database (INDD) exemplify how interconnected data repositories assist breakthroughs in neurodegenerative research [34].

An example of the importance of data sharing was the implementation of magnetic resonance imaging (MRI) as an AD diagnostic. While undergoing refinement, many neuroscience centers in the United States entered their data into the Alzheimer’s Disease Neuroimaging Initiative open-access database. This approach gathered AD imaging biomarkers across a continuum of older adults. Ten years after the project was launched, it was recognized as the starting point for more than 600 published AD-related papers [35]. Thus, global collaboration is a necessary entity to further AD research and is part of the journey to achieving health equity; all populations around the world must have equal opportunities to be in good health [36].

#### 3.1.2. Addressing the Pharmaceutical Challenge

Access to essential pharmaceuticals remains a challenge in LMICs, with over two billion people globally not having access to essential medications [23]. Structural and regulatory barriers, including patent protections, may delay the availability of more affordable generic alternatives [37]. These constraints contribute to inequities in drug access. Increasing competition and creating better pricing are necessary to close the drug gap [23,38]. Even with provisions such as the Trade-Related Aspects of Intellectual Property Rights (TRIPS) agreement allowing for the emergency production of generic drugs, resource limitations in LMICs may impede their distribution.

Thus, even if there were a ready cure for AD, it would not be accessible to everyone. Policy changes need to ensure accessibility and affordability prior to an AD cure being on the market. As mentioned with the COVID-19 vaccine rollout, pharmaceutical research and access predominately favor wealthier nations. The specific costs associated with developing AD drugs and conducting clinical trials are extremely high, and the drug market prices will only continue to increase [39]. Pharmaceutical companies and researchers need to expand their studies into LMICs; there may be faster recruitment and increased population diversity, allowing for a better understanding of AD [39,40].

#### 3.1.3. Expanding Diversity in Research

Increasing diversity in research, through different locations around the world, is critical in ensuring the generalizability of study results [41]. For example, both African American/Black and Hispanic/Latino populations are notably underrepresented in research studies and have an increased risk of being missed for an AD diagnosis [42,43]. There must be increased diversity across all levels of trial enrollment factors, such as, but not limited to, education, race, ethnicity, and socioeconomic status [44]. Amongst these factors, there may be underlying reasons why some groups experience cognitive decline faster or are more susceptible to AD development [43].

When addressing global commitments to healthcare delivery, there are societal factors that hinder access to AD clinical trials and which must be taken into consideration, including a lack of access to health services, such as living in an extremely rural area or experiencing poverty [45]. Matters are complicated further as caregivers are often included in AD clinical trial procedures, with many not being able to take a leave from their employment or having other responsibilities (e.g., young children). This makes it crucial that there is transparency in the clinical trial process, such as covering travel expenses or potential reimbursement for lost pay for caregivers or creating services that help those living with AD participate in a clinical trial (e.g., a transportation service). Furthermore, increasing trial awareness, in general, such as through marketing, is necessary to increase trial enrollment across a multitude of factors.

### 3.2. Diagnostic Development

Commitment: The creation of an affordable, accessible, and non-invasive diagnostic tool for AD that can be implemented worldwide.

**Figure 3:**
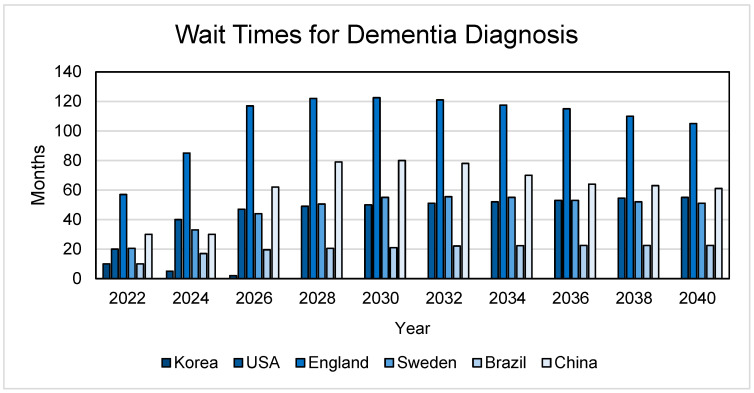
Wait times for dementia diagnosis. The wait times represent a referral to a dementia specialist and the assumption that a primary care physician has not made a dementia diagnosis. These studies involved examining the wait times for an appointment with a dementia specialist should these countries have access to disease-modifying AD treatments [52,53,54,55,56,57].

Advancements in AD diagnostics are crucial for early intervention and improved patient outcomes (more time to evaluate treatment/clinical trial options), thereby challenging the misconception that dementia is an inevitable consequence of aging [46,47]. There are probably more people with AD than estimates suggest; thus, early detection can assist in improving patient care, quality of life, and lengthening life expectancy [22,48]. Diagnostic methods have relied primarily on diverse techniques including cognitive assessments via neuropsychology tools often validated only in a limited range of languages and cultures; neuroimaging techniques such as MRI, computerized tomography (CT), and positron emission tomography (PET) for tau, β-amyloid and fluorodeoxyglucose; and cerebrospinal fluid sampling by lumbar punctures [22,49,50]. These methods are expensive and often inaccessible, as many LMICs have limited-to-no access to expensive machinery such as MRI scanners [51]. Likewise, a majority of these tests are only offered by dementia specialists, for whom there are lengthy wait times (see Figure 3) to obtain an appointment due to their limited number. Ongoing research into the development of cheap and accurate blood tests for amyloid and tau AD biomarkers needs enhanced support to facilitate their adoption on a global scale.

In addition, diversifying analytical approaches beyond β-amyloid detection could enhance diagnostic accuracy [58,59]. For example, an early diagnostic marker for AD may be enlarged glymphatic and associated perivascular spaces (ePVSs), which are visible on imaging (MRI); however, such techniques are expensive with reduced global availability [60]. Alternatively, an electroencephalogram (EEG) could possibly improve the availability of AD diagnostic procedures [61]; EEG is a non-invasive, low-cost and readily available tool that measures the electrical activity of the cerebral cortex, with the signals being correlated to different stages of AD [62]. Though lacking the structural sophistication of MRI, widely available diagnostic techniques, like EEG, need better study and implementation.

Oculomotor behaviours may be additional cost-effect biomarkers because they are linked to cognitive performance [61]. Visual gaze and pupil responses during short-term memory binding tests predict the presence of AD pathology as these elements rest on a network involving cortical and subcortical structures [61]. Proteins associated with AD accumulate in the eye; during AD pathogenesis, these proteins cause changes to retinal structure and vasculature, producing alterations that can be assessed as a non-invasive biomarker [63]. Visual disturbances may also hinder gait speed, as a decrease in walking speed is strongly associated with cognitive decline, and may be a cost-effective, easily implemented useful factor to include in dementia assessments [64].

The development of blood-based AD biomarkers (detecting analytes such as those involved in protein misfolding [e.g., amyloid-β, phosphorylated tau] or neuroinflammation) may emerge as potentially accessible, non-invasive and inexpensive screening tools [22,49]. For instance, a test measuring serum phosphorylated tau (p-tau) concentrations appears to be as accurate as cerebrospinal fluid biomarkers [65], and may be 90% accurate in identifying AD, compared to a specialist performing a cognitive evaluation or interpreting imaging, which is 63–73% accurate [66]. As shown in Figure 3, such biomarkers, if proven successful, could reduce wait times for expert-administered cognitive assessments by up to six years. However, these diagnostic tests hold promise only if made universally available, bridging the gap between wealthy nations and LMICs [22].

Additionally, although they constitute the time-honored diagnostic approach to AD, conventional cognitive testing likewise needs improvements; these tests are time demanding, laborious, language/culture specific, and repeated assessments allow patients to learn the test, a problem sometimes made worse by the sharing of test questions amongst patient families. Moreover, the tests need to take into consideration demographic factors, such as education, literacy, and cross-cultural variation [61]. Using artificial intelligence (AI) to conduct these tests could revolutionize cognitive assessments by enhancing test sensitivity across diverse populations [61].

### 3.3. Therapeutics Development

Commitment: Advance AD therapeutic development by focusing on the “ABCDE”s of drug discovery, exploring innovative and/or combination therapies with a focus on emerging or novel theories and approaches, and investing resources into the development of affordable and widely accessible therapeutics.

Advancing AD therapeutics demands embracing innovation and learning from both successes and failures. History has shown that pioneering discoveries, such as the serendipitous discovery of penicillin, may arise unexpectedly, indicating that a willingness to embrace new directions with experimentation and perseverance in drug development are needed. As research progresses, it has become evident that exploring new hypotheses beyond the traditional β-amyloid cascade hypothesis is undoubtedly necessary. Although β-amyloid aggregates have been central to AD research, their role in disease progression remains contested, and investigations into alternate pathways such as neuroinflammation, synaptotoxicity, and oxidative stress are needed to obtain a nuanced understanding of the disease [48,67,68,69]. An additional research direction to be investigated pertains to the underlying biological reason why more females than males have AD; a rigorous and fulsome exploration of this issue could alter treatment options or herald new paths to precision medicine [70].

#### 3.3.1. ABCDE Paradigm

Moving forward, a strategic approach represented by the “ABCDE” paradigm may enhance the likelihood of AD drugs progressing to clinical trials. This paradigm emphasizes accessibility (“A”), drug penetration across the blood–brain barrier (“B”), cognitive disease modification by curative, rather than symptomatic, agents (“C”), multi-target drugs that polypharmacologically bind to multiple complementary receptors (“D”), and environmental safety (“E”) as essential criteria for drug development [71]. Prioritizing these aspects streamlines drug design and ensures sustainability and a broader reach, which are particularly crucial elements for LMICs.

#### 3.3.2. Combination Therapies and Repurposed Agents

AD may require multiple pharmaceuticals given the pathogenic complexity of AD; combination therapies targeting multiple receptor-mediated mechanisms may offer effective treatment regimens for the disease [50]. Combining drugs such as memantine and donepezil has shown additive symptomatic improvements in individuals with AD [50,72]. From a disease modifying perspective, combining anti-protein misfolding therapies with agents targeting neuroinflammation might dramatically improve therapeutic strategies via either additive or synergistic effects [8]. However, despite such potential benefits, combination therapies come with their own regulatory approval hurdles that must be surmounted when attempting to confront the unique therapeutic challenges presented by complex, chronic neurodegenerative disorders such as AD.

Repurposing existing drugs already on the market could accelerate AD drug development. These agents have already passed toxicology assessments, having the potential to accelerate the AD drug development pipeline by three to five years [58]. Additionally, these agents may be more widely available to LMICs, as opposed to expensive novel molecules. However, despite their appeal, financial and regulatory challenges associated with repurposing compounds without strong patent protection remain significant barriers to developing therapies for multi-faceted and expensive disorders such as AD.

#### 3.3.3. Small Molecule Therapeutics

Pricing strategies for new pharmaceuticals have considerable influence on medication accessibility, often imposing costs that restrict access in LMICs or create financial emergencies for patients paying out-of-pocket [73]. This issue has gained considerable relevance in the realm of AD, given the recent focus on the development of biologics for AD indications; the initial price of aducanumab, a short-lived AD drug, exemplifies this issue. The initial annual pricing of aducanumab was set at USD 56,000—an amount that would have been prohibitive for LMIC market penetration. The price was subsequently decreased to USD 28,000 before the agent was removed from the market [35,74].

The commercial path of aducanumab is a cautionary tale regarding the worldwide accessibility of biologics aimed at treating AD [48,67,74]. LMICs may not be able to afford expensive treatment options such as biologics (e.g., lecanemab and donanemab) [75]. The continued development of biologics as putative therapeutics for AD necessitates the concomitant implementation of policies for developing less costly biosimilars and other cost-containment strategies to enable improved global availability of potentially life-saving agents.

An alternative solution involves small molecule therapeutics (molecular weight < 650 g/mol). These low-molecular-weight entities typically exhibit cost-effective production, prolonged shelf-life, and ease of distribution, storage, and administration [76]. Furthermore, small molecules are more accessible to LMICs and remote areas, as these optimal qualities allow the molecules to be administered orally without hospital facilities. By comparison, biologics typically require the patient to travel to a hospital or clinic setting; not only is this geographically limiting, but also creates a financial challenge in transportation to these sites [77]. Ultimately, the present urgency for diverse and all-encompassing thinking in AD research stems from the current lack of effective treatments that stop or reverse AD’s relentless pathology [67]. Global investment in therapeutic development, from time, personal commitment, and financial resources, not only holds the promise of transformative treatments but also potentially alleviates future worldwide healthcare burdens associated with AD.

### 3.4. Risk Factor Management (5 Preventions)

Commitment: Establishing a person-centered and adaptable strategy for improving brain health globally, while mitigating the risk of AD by promoting risk-prevention strategies such as the SHIELD Strategy (sleep, head injury prevention, exercise, learning, and diet).

Given the struggles to devise curative therapies for AD, pursuing approaches that reduce the likelihood of developing dementia is prudent from a global health perspective. How the brain ages is directly influenced by its past, present, and even future exposures: its exposome. Some studies have suggested that approximately 33–45% of AD cases can be prevented by risk factor mitigation [78,79]. The influential 2020 Lancet Commission identified twelve modifiable risk factors for dementia: diabetes, air pollution, physical inactivity, social isolation, depression, smoking, obesity, alcohol abuse, hypertension, traumatic brain injury, hearing loss, and lower education. In 2024, an update added vision loss and hypercholesterolemia [80,81]. With risk factors in mind, a recent clinical trial involved participants diagnosed with AD who underwent intensive lifestyle changes (e.g., eating a healthy vegan diet, daily exercise, and stress management). Among the twenty-four patients in the intervention group, ten reported improvements in their cognitive function; no patients in the control group (no lifestyle interventions) experienced any cognitive improvement [82]. Thus, risk factor reduction may be able to slow cognitive progression (and perhaps be preventative in younger populations).

**Figure 4:**
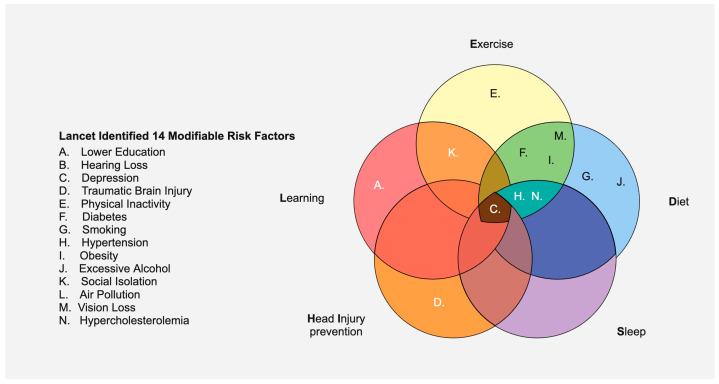
The five proposed areas of focus that provide protection against the majority of the fourteen Lancet-identified risk factors for dementia. Hearing loss (“B”), air pollution (“L”), and vision loss (“M”) were omitted, as there is currently no direct evidence that SHIELD could be preventative for these factors.

An anti-dementia public health paradigm built on 12–14 risk factors, though unquestionably valuable, is complex and thus challenging to market and socialize. There is a need for simplified branding of this message to facilitate uptake and marketing with mass appeal. Inspired by this, we identify five areas of focus to decrease the risk of dementia: sleep, head injury prevention, exercise, learning, and diet (SHIELD Strategy). These five elements overlap, such that one action is protective against multiple identified risk factors, providing multi-faceted protection (see Figure 4); the SHIELD Strategy is an attempt to take the 14 modifiable risk factors for AD and repackage them into a less intimidating five-factor approach. Arguably, the person who adopts the SHIELD Strategy for lifestyle changes may reduce their risk of AD and may mitigate cognitive decline if the disease develops.

#### 3.4.1. Sleep

Investment in adequate sleep may help prevent AD and is an accessible target for worldwide focus [83]. During sleep, amyloid-based proteotoxins contributing to the formation of AD plaques are removed from the brain [84,85]. Chronic sleep deprivation, a global issue, leads to increased soluble β-amyloid levels, which can contribute to characteristic plaque development [86]. Thus, minimizing sleep debt is critical to give the brain a chance to rid itself of proteopathic biomolecules. Moreover, rest provides the energy needed to be physically active, the focus required for educational endeavours, is protective against depression, and helps an individual follow a healthy diet (decreases the risk of over-eating). Thus, sleep decreases the risk of a multitude of dementia risk factors, all of which directly or indirectly aid in preventing AD development [84,87]. Strategies for global brain health require that we keep sleep in mind.

#### 3.4.2. Head Injury Prevention

Directly protecting the head is essential to healthy aging, both cognitively and physically. Concussions or mild to traumatic brain injuries (TBIs) increase the risk of developing dementia, making it imperative to take action to reduce the risk of these injuries [88]. Of additional concern are TBIs stemming from intimate partner violence (IPV) or domestic violence, and the substantial risk of developing dementia in the future [89]. Since many head injuries are accidental (e.g., falls, motor vehicle collisions), ensuring there is information on a safe recovery process to prevent the risk of additional dangerous head injuries is obligatory. For example, with athletic activities, there must be immediate removal from play if a concussion is suspected. Similarly, the use of appropriately designed helmets in athletic activities, when appropriate, must be enforced, as these reduce the risk of a concussion (or concussion severity) by minimizing head acceleration upon impact [90]. Protecting the head physically allows for healthier aging—allowing for better memory and focus. SHIELD is an important part of our defensive armour against AD.

#### 3.4.3. Exercise

Physical inactivity is a brain health issue. In North America, in which one in three adults live sedentary lifestyles, lack of exercise directly contributes to 21% of AD cases [91,92]. Consistent exercise promotes hippocampal growth, enhancing cognitive and cortical function to protect against AD-related atrophy [93,94]. Exercise can upregulate brain-derived neurotrophic factor (BDNF), which assists brain repair while counteracting anxiety and depression [92]. Specifically, aerobic exercise enhances synaptic plasticity in the frontal and parietal gray matter by increasing blood supply to these regions, offering neuroprotection [94]. These elements contribute to improvements in episodic memory, executive function, attention, and processing speed, decreasing the risk of cognitive impairment [95]. The benefits of exercise extend across multiple other risk factors including obesity, hypertension, hypercholesterolemia, and depression; people who exercise regularly are less likely to smoke, and exercise can be a social activity. Regular participation in social leisure activities during middle age reduces dementia risk by close to 50% [96]. Even a small commitment, such as one hour of aerobic activity with peers per week, decreases the risk of dementia [97]. Fitter is smarter for brain health.

#### 3.4.4. Learning

Learning and education are protective against AD; individuals with lower education levels (i.e., not finishing secondary school) face a 40% greater risk of dementia [98]. Efforts to increase literacy rates, school enrollment, and educational access, especially for females in LMICs, are crucial. Educated people are less likely to smoke or be obese; education provides the opportunity to socialize and make long-lasting connections, both of which decrease loneliness, reduce stress, and protect against AD [99,100].

Education contributes to building a cognitive reserve. A cognitive reserve is defined by an increase in the complexity of neuronal connections, so when one neuronal path is destroyed due to AD, there are other pathways through which the signal can access the information [92]. Activities such as daily puzzles improve cognitive function and reduce brain atrophy in individuals with mild cognitive impairment. Learning a musical instrument decreases the risk of dementia by 64% and contributes to the building of a cognitive reserve, hence having music classes offered through schools is important, according to the World Alzheimer’s Report 2023. An example of the importance of a cognitive reserve was demonstrated during the “Nun Study”, in which 8% of participants with a severe spread of AD pathology remained at the same level of cognitive function as those without any plaques and tangles [101,102].

Rejecting ageism (the assumption that people cannot do certain tasks or activities because of their age) aids cognitive reserve protection. A common form is “elderspeak”, in which specific words are changed when communicating with an older person, believed to help their understanding of the matters being conveyed to them. This is patronizing and can precipitate communication breakdown, increasing cognitive decline as one continues to age [103]. Addressing and making efforts to avoid ageism practices will help people feel more positive about aging, and by doing so, their performance will improve on memory tests, they may heal from illnesses or injuries faster, feel happier, and have the potential to live longer [103].

Another example of building cognitive reserve is through multilingualism [104]. Connections from the vision center to the language comprehension centers in the brain receive increased blood supply, increasing angiogenesis to create protection associated with cognitive reserve [92]. In particular, bilingualism can delay the onset of dementia symptoms by 4–5 years compared to monolingualism [104,105]. This is a sevenfold increase in protection against cognitive impairment, but the languages spoken need to be frequently practiced to achieve cognitive protection [92]. Thus, school is a tool for prolonged brain health.

#### 3.4.5. Diet

Though learning and exercise are crucial areas of focus, they cannot compensate for a poor diet. The Mediterranean-DASH Intervention for Neurodegenerative Delay (MIND) diet is recognized for its role in brain and cerebrovascular health, as well as being affordable [84]. The MIND diet consists of a typical healthy diet, with the majority of intake being from fruits, vegetables, legumes, whole grains, poultry, and fish, rather than from red meat. There are very limited amounts of alcohol, saturated fat, simple sugars, and no consumption of tobacco/nicotine products. Moreover, the financial cost of the MIND diet is less compared to the economic cost of AD. The caloric intake and protein content of legumes and beans, a large portion of the diet, are nearly identical to those of red meat. An additional benefit comes from legumes and beans containing less fat, being less expensive, and having more fiber than red meat [106].

Adult participants in a long-term study adhering to the MIND diet had cognitive scores of those seven years younger [84]. Diet is protective for health; Nigeria has the highest prevalence of APOE ε4, but fewer AD cases than Indianapolis, USA, where there are five times as many cases but fewer genetic factors. This can be attributed, in part, to the healthier Nigerian diet, which is low in saturated fat, and that a majority of Nigerians live a more active life [84,107]. In general, a well-balanced diet is also protective against depression, hypertension, obesity, and provides energy necessary for physical activity and education, all of which lower the risk of dementia [108]. The world grows enough food—small investments in the equitable distribution of nutrients will enable major brain-health dividends globally: nutrition for neurons is food for thought.

### 3.5. Improved Care for People with AD Worldwide

Commitment: Improve care for those with AD on a global scale by dismantling the stigma surrounding the disease and changing how we respond to it. This can be achieved through increasing education and awareness about dementia through targeted campaigns and programs.

#### 3.5.1. Stigma

AD remains one of the most stigmatized health conditions worldwide. Harmful phrases such as “the long goodbye”, “death before death” and “the funeral that never ends” perpetuate a stereotype that individuals with dementia experience a form of premature death, with their cognitive decline overshadowing their lived experience [109,110]. Despite the disabling memory loss associated with dementia, these changes occur gradually rather than suddenly [111]. Acknowledging and fulfilling emotional needs that remain despite cognitive decline will significantly improve the care provided to individuals with dementia [109,112]. Many people with AD continue to lead fulfilling lives post diagnosis, with some living more than two decades [113]. Therefore, it is crucial to address and rectify the sometimes malignant social positioning associated with dementia, especially given that the last four Alzheimer’s Disease International World Alzheimer’s Reports (2019–2023) have shown minimal improvement in societal attitudes.

A significant aspect of the stigma involves avoidance. Although dementia is not contagious, there exists a pervasive fear that proximity to someone with dementia might increase one’s susceptibility to the disease [110]. Upon diagnosis with the disease, for example, half of Canadians believe their quality of life would decrease almost immediately, and 25% believe their life would be over, according to the Alzheimer’s Society of Canada. This fear often leads to the social exclusion of individuals with dementia, causing their pre-diagnosis identity to be overshadowed by their current condition. While it is understandable to use aversion as a coping mechanism, this response can exacerbate the cognitive decline of individuals with AD through social isolation [114]. It remains essential to prioritize empathy over fear, as these social connections are arguably more important now than ever.

Moreover, there is a tendency to assume that communication with individuals with dementia will be challenging. Cognitive abilities may fluctuate as dementia progresses, leading to days of difficult conversing, whereas on other days the person may converse like their pre-diagnosed self. Patience and accepting the communication efforts of individuals with dementia are vital. Techniques from improvisational theater, such as the “yes, and….” approach, can be effective for those with advanced-stage dementia [115,116]. This method involves building on the person’s communication attempts rather than correcting them, thereby encouraging individuals with dementia to express their needs and emotions, and maintain connections with their loved ones. While immediate changes to health services and the development of disease-modifying therapeutics may be beyond reach, especially in LMICs, we can control our personal responses to dementia. A person’s identity is significantly shaped through their interactions with others—an important reminder when managing a neurodegenerative disease that causes a progressive loss of independence.

#### 3.5.2. Educational Programs

Education fosters understanding, which is how perceptions and stigma of dementia will be changed. Enhanced awareness of AD can lead to improved care for individuals living with the disease [117]. Public education campaigns, including social media outreach and advertisements, can play a significant role in disseminating information about dementia and effective communication strategies.

Several countries have implemented programs focusing on inclusivity for people with dementia. In Bruges, Belgium, a red sticker on the window of a shop indicates that the shopkeepers are trained to effectively communicate with people with cognitive impairment [35]. While these specific services may be difficult to implement, widespread training in communicating with someone with cognitive impairment is attainable and has societal benefits. Creating an online, multilingual module-based training program on communication with individuals with cognitive impairments could be a viable option.

Additionally, it is essential to include individuals with dementia in the development and delivery of these proposed educational programs [109]. For example, the Dementia Alliance International presented at the 12th Conference of State Parties on the Convention on the Rights of Persons with Disabilities (COSP12) to represent dementia as a disability—presented by people with dementia. This was the first time a major conference had this initiative. Events similar to these provide invaluable knowledge, as these individuals have lived experiences that should be the basis of forming educational content.

Extending these educational programs to include caregivers will improve care for individuals with AD, since as dementia progresses, authority shifts, and the caregiver assumes more responsibility [35]. This responsibility is often associated with high levels of stress and burnout, which may lead to a caregiver inadvertently directing their frustrations against those under their care [118]. These programs can teach coping mechanisms, decreasing the risk of abuse or neglect of the person with dementia [119]. Through better education on dementia, from aspects of the disease itself to communication methods for someone with advanced-stage dementia, we can eradicate the stigma and improve care worldwide.

## 4. Conclusions

### 4.1. Global Diseases Need Global Solutions

Alzheimer’s Disease extends far beyond the individual whose brain is afflicted with the characteristic plaques and tangles; AD influences the lives of all those connected to the person; it affects families, friends, communities, and societies, on a worldwide scale. AD is associated with relentless cognitive decline, which exacts a grim toll, emotionally and physically. Presently, millions globally endure the direct and indirect repercussions of this disease, which impact these individuals and their social networks in many and varied pervasive ways. Beyond the staggering numbers, the AD global health emergency exceeds public health boundaries—it is an exigent humanitarian issue. The widespread nature of AD thus constitutes a need for urgent intervention. To safeguard our aging populations, there must be a coordinated global response. Solutions to this escalating global emergency are not merely a necessity, but a moral obligation, demanding immediate and coordinated action on an international scale.

### 4.2. Call to Action

To address the AD emergency with the urgency it demands, a comprehensive and coordinated global response is imperative.

1. Alzheimer’s research must be redefined as a global enterprise requiring worldwide participation in all aspects, recognizing that the challenges are irrespective of borders and necessitate a unified effort and substantial investment of time and resources.
2. Non-invasive, accessible, and affordable diagnostic tools must be urgently developed, as they are crucial for early detection and improving patient outcomes worldwide.
3. Alzheimer’s therapeutics discovery must be advanced through cutting-edge research into globally available therapies by pursuing innovative and pioneering theories to enable the identification of disease-altering treatments.
4. There must be promotion of strategies such as the SHIELD five dementia-preventative lifestyle modifications—head injury prevention, regular physical exercise, education, a balanced diet, and adequate sleep—which can mitigate an influx of cases later in life.
5. The stigma associated with AD must be eradicated through extensive educational programs to improve the care for those living with the disease in all areas of the world.

By committing to these strategic actions, we can drive a unified and impactful effort to address AD with the scale and attention it requires, all whilst continuing to work towards a cure.

## Data Availability

The original contributions presented in this study are included in the article. Further inquiries can be directed at the corresponding author.

## Abbreviations

The following abbreviations are used in this manuscript:

MDPI	Multidisciplinary Digital Publishing Institute
DOAJ	Directory of open access journals
AD	Alzheimer’s disease
ADRD	Alzheimer’s disease and related dementias
CDC	Centers for Disease Control and Prevention
WHO	World Health Organization
LMICs	Low-and middle-income countries
CSF	Cerebrospinal fluid
GAAIN	Global Alzheimer’s Association Interactive Network
INDD	Integrated Neurodegenerative Disease Database
MRI	Magnetic resonance imaging
TRIPS	Trade-Related Aspects of Intellectual Property Rights
CT	Computerized tomography
PET	Positron emission tomography
ePVS	Enlarged glymphatic and associated perivascular spaces
EEG	Electroencephalogram
AI	Artificial Intelligence
SHIELD	Sleep, Head Injury prevention, Exercise, Learning, and Diet
TBI	Traumatic brain injury
IPV	Intimate partner violence
BDNF	Brain-derived neurotrophic factor
MIND	Mediterranean-DASH Intervention for Neurodegenerative Delay
